# Co-designing a psychoeducational intervention for FCs of institutionalized older adults : a participatory double diamond approach

**DOI:** 10.1186/s12877-026-07398-7

**Published:** 2026-04-06

**Authors:** Souad Meziane-Damnée, Anne-Sophie Rigaud, Catherine Bayle, Matthieu Piccoli, Lauriane Blavette, Sébastien Dacunha, Hermine Lenoir, Maribel Pino

**Affiliations:** 1 Université Paris Cité, INSERM UMR-S 1144, OTEN, Paris, France; 2 Service Gériatrie 1&2, Centre Mémoire de Ressources et Recherches Ile de France, AP-HP, Paris, Paris, F-75013 France; 3 Centre d’Expertise National en Stimulation Cognitive (CEN STIMCO), 54 rue Pascal, Paris, 75013 France

**Keywords:** Dementia, Family caregivers, Institutionalization, Psychoeducational intervention, Participatory design, Geriatric care, Qualitative research

## Abstract

**Background:**

The transition of a relative with dementia into long-term care is a critical and emotionally charged period for family caregivers, often marked by guilt, loss of role, and strained relationships with care teams. While psychosocial interventions can mitigate these challenges, few are co-designed with both caregivers and professionals to ensure contextual relevance.

**Methods:**

Using the Double Diamond design framework, we conducted a four-phase co-design process in geriatric institutions in Île-de-France: *Discover* (inductive analysis of 14 interviews with family caregivers within 6 months of admission), *Define* (deductive validation and prioritisation of needs through focus groups with caregivers and professionals), *Develop* (collaborative design of a psychoeducational intervention), and *Deliver* (pilot testing with eight family caregivers and six professionals, followed by focus group feedback). Analyses alternated inductive and deductive approaches according to phase objectives.

**Results:**

The needs assessment identified ten thematic domains, including emotional ambivalence, redefinition of the caregiving role, communication breakdowns, and support gaps. These informed the creation of a seven-session group programme with two individual sessions, addressing dementia knowledge, role adaptation, communication skills, nutrition, and end-of-life planning. In the pilot phase, feasibility was high, with no dropouts and sessions deemed appropriate in number, duration, and content. Acceptability was strong, although the end-of-life module was emotionally challenging. Caregivers reported improved understanding of institutional care, enhanced communication with staff, reduced anxiety, and strengthened peer support. Professionals noted improved relationships with families and reduced conflict over day-to-day care issues. Feedback led to refinements, including short summary videos for each session, reorganisation of sensitive modules, and integration of practical communication tools in the final session.

**Conclusions:**

This co-designed psychoeducational intervention addresses a critical gap in supporting family caregivers during the transition to institutional dementia care. Grounded in lived experiences and professional insights, it demonstrates high feasibility and acceptability, with promising benefits for caregiver well-being and family–staff collaboration. An efficacy trial is warranted to assess its impact on caregiver outcomes and institutional climate.

**Trial registration:**

NCT05651555.

**Supplementary Information:**

The online version contains supplementary material available at 10.1186/s12877-026-07398-7.

## Introduction

The institutionalization of an older adult with a major neurocognitive disorder such as Alzheimer’s disease marks a profound transition in the caregiving trajectory, reshaping rather than ending the caregiving role. While placement in a geriatric facility may alleviate the physical demands of daily care, it frequently introduces new psychosocial challenges [1] including guilt, grief, role ambiguity, anxiety, and a perceived loss of involvement in the care process [2–4]. Families are required to shift from hands-on caregiving at home to a role centred on advocacy, emotional presence, and adaptation to institutional structures [5, 6].

Family caregivers (FCs) remain key stakeholders in the care process even after a relative with dementia enters an institutional long-term care setting, maintaining their involvement through visits, monitoring of care quality, and negotiation of their role within facility routines. Despite this engagement, they often face barriers to effective communication with care teams—such as unclear information, limited dialogue, and inconsistent responsiveness—along with inadequate orientation to institutional practices and insufficient emotional support [6–8]. These difficulties are magnified in the context of dementia, where cognitive decline alters interpersonal dynamics, and the progressive nature of the disease generates uncertainty and anticipatory grief [7]. Despite their ongoing involvement, many FCs report feeling sidelined by institutional cultures that prioritise clinical workflows over family inclusion [1, 9].

Psychoeducational interventions for FCs who place their family members in long-term care have been developed to address these challenges, aiming to strengthen their coping strategies, enhance their knowledge, and build their confidence through information, skills training, and emotional support [10–18]. For example, Gaugler et al. (2008) developed the *Residential Care Transition Module*, an individualised psychoeducational intervention delivered after nursing home placement that integrates education about institutional care with emotional support and problem-solving strategies [12]. Similarly, Schulz et al. (2014) evaluated an individual-based psychosocial intervention aimed at supporting family caregivers through education on long-term care processes and resident trajectories, guidance on advance and end-of-life care planning, and targeted support for caregivers’ emotional difficulties [14]. In contrast, Ducharme et al. (2005) described a group-based psychoeducational programme structured around six key domains, including maintaining a meaningful relationship with the institutionalised relative, communicating effectively with healthcare staff, managing emotional distress, coping with cumulative losses and preparing for bereavement, mobilising social and community resources, and reorganising personal life with attention to self-care following institutionalisation [10]. Finally, Paun et al. (2015) proposed a group-based programme using guided discussions to strengthen caregivers’ knowledge of dementia and to develop skills in communication, conflict resolution, and chronic grief management [15]. Such programmes often adopt a holistic approach, addressing both the emotional and practical dimensions of informal caregiving after a relative’s institutional placement. Reported effects are mixed: while some studies show reductions in guilt, psychological distress, and perceived burden, improvements in depression and satisfaction with care are less consistently observed [17, 18]. Moreover, few interventions for FCs have been developed using participatory approaches that incorporate both their lived experiences and the expertise of professionals in geriatric care institutions. The absence of such co-design processes can limit an intervention’s contextual fit, cultural relevance, and overall acceptability.

The present article describes the development of a psychoeducational program for FCs of relatives with dementia who are undergoing the transition from home to institutional long-term care settings. The program was theoretically grounded in Lazarus and Folkman’s transactional model of stress and coping [19], which conceptualises stress as arising when perceived demands exceed available coping resources. Cognitive appraisals of these demands, together with the individual’s perceived ability to manage them, shape emotional and behavioural responses. Guided by this framework, the program was designed to enhance FCs resilience by increasing understanding of dementia, strengthening emotional support, and fostering adaptive coping strategies.

Participatory co-design approaches offer distinct advantages in the development of programmes for FCs. Engaging both FCs and professionals in the design process ensures that intervention content is firmly grounded in lived experience, responsive to practical constraints, and positioned for successful implementation in real-world settings [20]. Such collaboration not only enhances the programme’s relevance and acceptability but also fosters trust and partnership between FCs and professionals in advance of its delivery [21, 22].

This study describes the development of a psychoeducational programme co-designed with FCs and healthcare professionals to support the transition of older adults with dementia into institutional long-term care. The process employed the Double Diamond model—an iterative, four-phase framework (*Discover*, *Define*, *Develop*, *Deliver*) developed by the UK Design Council [23, 24], that promotes iterative exploration, problem definition, solution generation, and implementation [23, 24]. Building on this foundation, the present work aimed to co-design, develop, and pilot-test the first version of a brief, adaptable, and scalable psychoeducational intervention tailored to the needs of FCs during the transition to institutional care.

## Methodology

### Context of the study

This study was conducted between November 1, 2021, and December 30, 2022, in Paris, France. The decision to co-design a psychoeducational programme emerged from repeated observations made by the mobile geriatric team of Broca Hospital (AP-HP), which systematically collected feedback from nursing home professionals and FCs during its coordination and support missions. These consultations consistently highlighted recurring challenges encountered by FCs of individuals with dementia during and after a loved one’s admission to institutional long-term care. In response to these identified needs, the mobile geriatric team led an interdisciplinary co-design process involving four physicians, three psychologists, and one researcher—all with expertise in dementia care—together with input from the nursing home professionals. The resulting programme was specifically developed to support family caregivers during the critical transition period following the institutionalisation of their relatives.

### Participants

Professionals and FCs from geriatric care institutions were recruited in collaboration with seven participating facilities. Invitations containing a study overview and researcher contact information were distributed via email and telephone through the institutions. Eligibility criteria for FCs included being the primary family contact of an older adult institutionalised, recently admitted (within the previous six months) to a long-term care facility, being over 18 years of age, and fluent in French. Professionals were eligible if they were directly involved in patient and family care within institutional settings.

Except for members of the co-design team, participants differed across all phases of the project, ensuring that each stage incorporated new perspectives and minimised overlap in individual experiences. This approach ensured that both lived experience and professional perspectives informed the needs assessment, solution generation, and definement of the intervention. No formal sample size calculation was performed for this study. Our recruitment method can be defined as convenience sampling as we included professionals and FCs who were available to participate in the study. Our interviews allowed us to reach data saturation. A total of 62 individuals—45 FCs and 17 healthcare professionals—were recruited through seven nursing homes in Paris, all providing care to residents with a diagnosis of dementia. Recruitment was conducted in collaboration with staff from these facilities. Fifty-two FCs were invited to participate; seven declined, citing time constraints or lack of interest. The final sample comprised 45 FCs (37 women, 8 men) .

In parallel, 24 healthcare professionals representing various roles within geriatric institutions (psychologists, physicians, nurses, and nursing assistants) were invited to participate. Seven declined, also due to time constraints. The final professional sample consisted of 17 participants (13 women, 4 men).

### Co-design procedures

**Fig. 1:**
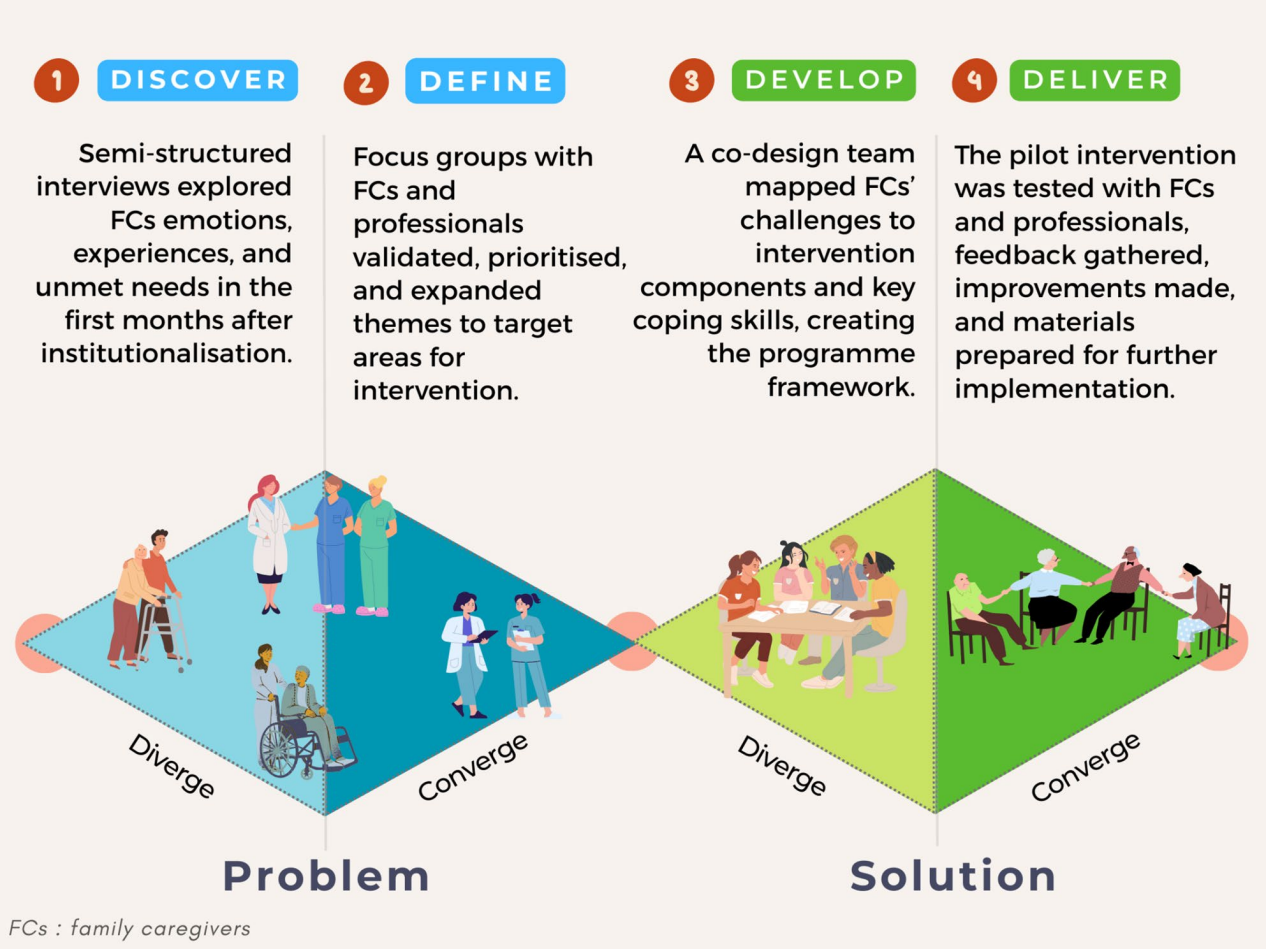
Co-design process for developing a family caregiver support intervention: double diamond framework

The co-design process followed the four phases of the Double Diamond model—*Discover*, *Define*, *Develop*, and *Deliver*—a human-centred design framework developed by the UK Design Council [23, 24], which alternates between divergent and convergent thinking. In divergent phases (*Discover* and *Develop*), participants generate a broad array of ideas, needs, and possibilities, fostering creativity and inclusive perspectives; in convergent phases (*Define* and *Deliver*), these ideas are critically evaluated, prioritised, and defined into actionable solutions. Active involvement of FCs and professionals would be expected to keep the intervention grounded in lived experience while aligning with practical and organisational requirements. This structured yet flexible co-design approach aligns with other health-related applications, for example, Banbury et al. [22] used the Double Diamond to develop a telehealth peer support programme for dementia FCs, and Johnson et al. [25] applied it to create a digital, family-led delirium prevention intervention in critically ill patients, demonstrating its adaptability across care contexts. The model is valued for its clarity, adaptability to different contexts, and emphasis on balancing divergent thinking (broad exploration of ideas and needs) with convergent thinking (focused definition of solutions). It fosters active stakeholder engagement throughout the process and supports the integration of multidisciplinary perspectives into practical, implementable outcomes. In this study, the model provided a structured pathway for involving family caregivers and institutional professionals at multiple stages. The process combined qualitative data collection through semi-structured interviews and focus groups with participatory validation workshops, ensuring that the emerging intervention was grounded in the lived experiences of caregivers and the practical realities of professional practice (Table 1). Fig. 1 illustrates the co-design process used to develop the family caregiver support intervention, structured according to the Double Diamond framework encompassing four phases: Discover, Define, Develop, and Deliver.

**Table 1:** Phases, data sources, analytic approach, and outputs

Phases	Data Source	Analytic Approach	Output
Discover	Individual semi-structured interviews with FCs	Inductive qualitative content analysis	Emerging thematic framework of caregiver needs and experiences
Define	Focus groups with FCs and professionals	Deductive qualitative content analysis (using framework from Discover phase)	Validated and expanded thematic framework; priority areas defined
Develop	Co-design workshops with co-design team and FCs	Collaborative synthesis of themes into programme components with iterative team review.	Structured psychoeducational programme with session plans, facilitator guidance, and supporting materials
Deliver	Program sessions with FCs and professionals Focus groups with FCs and professionals	Inductive qualitative content analysis	New insights, unanticipated suggestions, and final refinements to the programme

In the “*Discover”* phase, the aim was to explore the lived experiences, emotions, and unmet needs of FCs during the early stages of institutionalisation. Semi-structured interviews were conducted with FCs of older adults who had been admitted to geriatric institutions within the previous six months, a period chosen to capture experiences and perceptions while the transition was still recent (Additional File 1). The interviews focused on emotional experiences, changes in the caregiving role, perceptions of institutional staff, and support needs.

In the “*Define”* phase, findings from the individual interviews were consolidated and defined. Focus groups with FCs were conducted to validate and deepen the themes identified during the *Discover* phase, allowing participants to confirm, prioritise, and expand upon emerging issues (Additional File 2). Additional focus groups with geriatric care professionals were held to validate these themes from clinical and institutional perspectives and to help identify domains requiring targeted intervention. 

In the “*Develop”* phase, the co-design team systematically translated the identified themes into a structured analytical framework. This framework, presented as a detailed table, mapped family caregivers’ challenges to targeted intervention components and delineated the specific skills required to foster effective coping. This framework served as the foundation for designing the full programme, ensuring a clear correspondence between the needs identified in earlier phases, the targeted content, and the planned skill-building activities. A group of FCs contributed to the framework’s development, drawing on their lived experience and practical expertise to validate the relevance and feasibility of each proposed component.

In the “Deliver” phase, the first version of the intervention was pilot-tested with a group of FCs and geriatric care professionals. Participants completed the full programme and provided feedback after each session. Two final group discussions, one with the family caregivers and one with the professionals, were held to assess the intervention’s overall feasibility, acceptability, and clarity, and to identify its key benefits along with suggestions for improvement (Additional File 3). At the end of this phase, the co-design team consolidated all feedback, made targeted structural and content adjustments, developed a detailed facilitator manual, and prepared the methodological framework for the forthcoming feasibility study.

### Study instruments and intervention development process

#### Interview and focus group

Semi-structured guides for interviews and focus groups with FCs and professionals (Phases Discover, Define, and Deliver) were developed using a multi-step process to ensure question clarity and content validity. First, SDac (psychologist) and LB (researcher) drafted initial questions based on existing literature on FCs’ unmet needs and psychoeducational interventions. Second, the guides were reviewed by two additional experts (MPin and ASR) to assess clarity, relevance, and coverage. Third, a pilot phase with test interviews involving health professionals (HL and MPic) was conducted to identify and revise ambiguous items. The core themes of the final guides are presented in Additional Files 1–3.

Each interview or focus group was conducted in a quiet, private meeting room within the participating facility to ensure confidentiality and minimise disturbance. A digital audio recorder was used to capture all sessions for verbatim transcription, with participants’ consent. Printed copies of the interview or focus group guides were available to facilitators to ensure consistency in question delivery, and printed consent forms were used for participant enrolment.

#### Collaborative workshops for programme development

In the Develop phase, collaborative workshops were conducted with the co-design team and a group of family caregivers to translate thematic findings into a structured analytical framework. Participants reviewed and defined a draft mapping of caregivers’ challenges to targeted intervention components and associated skill-building activities.

Workshops took place in meeting rooms within the participating institution and were designed to promote open discussion and collaborative problem-solving. Materials included printed summaries of the thematic analysis, the draft framework, sticky notes for individual contributions, markers, and a projector for displaying and editing the content collectively.

The procedure followed a structured creative workshop format:

1. Presentation of findings from earlier phases to provide a common reference point.
2. Brainstorming and discussion in which participants proposed definements, shared perspectives, and challenged assumptions.
3. Collective validation of proposed changes, facilitated to ensure balanced participation.
4. Real-time documentation by the facilitators, noting both agreed revisions and items for further review.

Through guided discussion and iterative feedback, the group validated the relevance, feasibility, and clarity of each proposed component, ensuring alignment with the needs identified in earlier phases. This process concluded with the creation of the initial version of the psychoeducational programme.

#### Pilot delivery of the psychoeducational programme

**Fig. 2:**
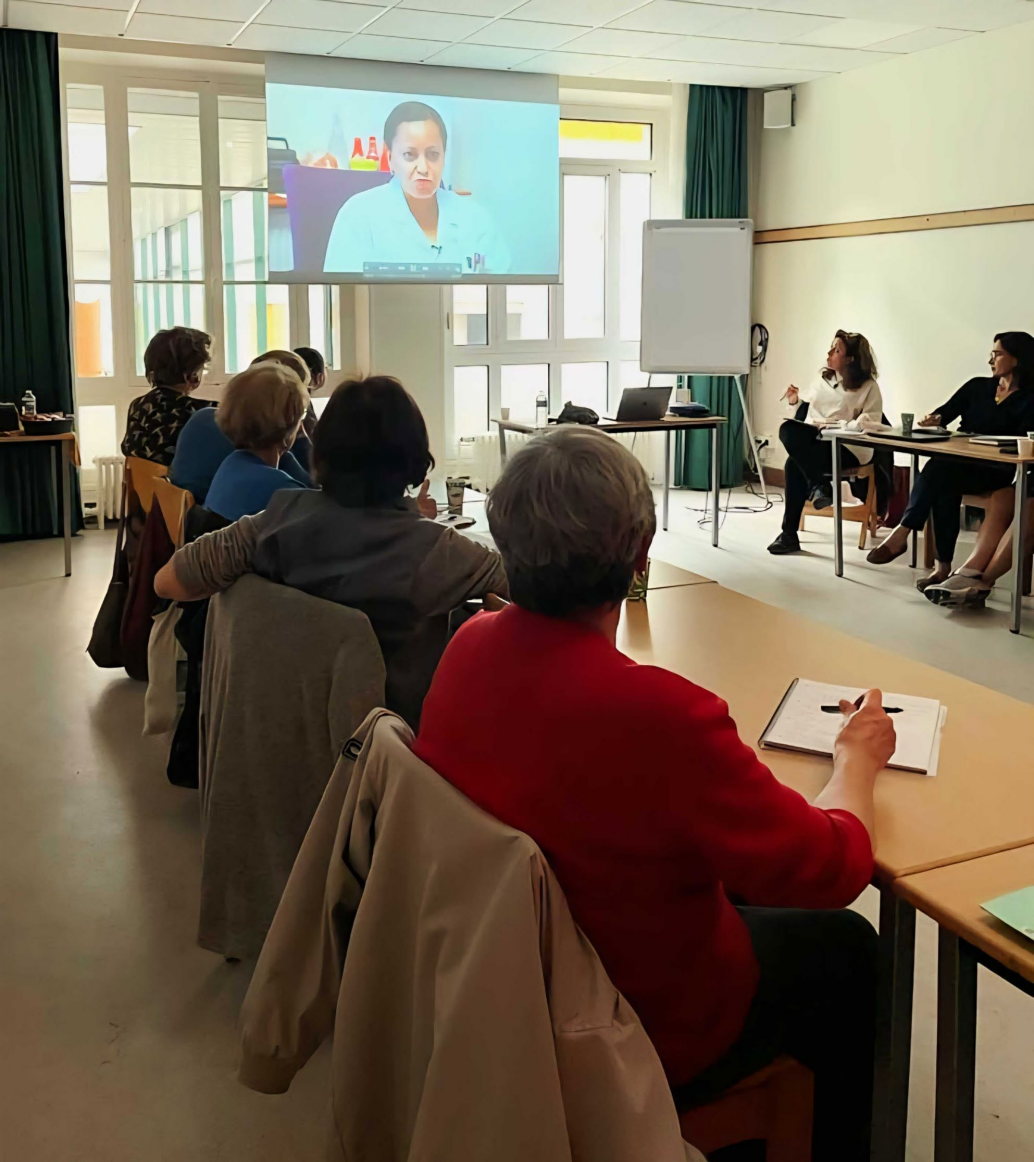
Group session of the psychoeducational program

In the *Deliver* phase, the initial version of the psychoeducational programme was implemented in pilot sessions with FCs (Fig. 2), held on-site at one of the participating facilities in a quiet, comfortable room. Sessions lasted approximately 90 min and were facilitated by members of the co-design team, following the structured content and activities developed in earlier phases.

Materials included printed handouts, PowerPoint slides projected during each session, and session outlines for facilitators. All sessions were audio-recorded with participants’ consent to ensure accurate capture of discussions for later analysis.

A typical session followed a consistent structure:

1. Welcome and introduction: informal greeting, recap of previous session, and presentation of the day’s topic.
2. Thematic input: facilitator-led presentation supported by PowerPoint slides, providing key concepts, examples, and practical tips.
3. Interactive discussion: guided group conversation and Q&A, encouraging participants to share personal experiences and reflect on the presented content.
4. Practical exercises: role-plays, case discussions, or problem-solving activities to apply the concepts in realistic scenarios.
5. Wrap-up and take-home messages: summary of key points, distribution of any supplementary materials, and an invitation for participants to note questions or concerns for future sessions.

The pilot delivery aimed to assess the programme’s feasibility, clarity, and acceptability in a real-world institutional context. The final contents of the pilot programme are presented in the Results section.

### Data collection

Two members of the co-design team (SDam and CB) conducted all face-to-face interviews and focus groups in the participating institutions. Neither had any prior relationship with participants. Before participation, individuals received detailed information about the study’s objectives and procedures and provided written informed consent. They were reminded of their right to withdraw at any time without justification.

Anonymity and confidentiality were ensured, with only participants and researchers present during sessions. All interviews and focus groups were audio-recorded with consent and anonymised during transcription; no field notes were taken. Each participant attended only one interview or focus group, and transcripts were not returned for feedback or revision. These procedures were applied consistently across all phases of the Double Diamond co-design process to maintain methodological coherence and data quality.

### Data analysis

All interviews and focus groups were audio-recorded and transcribed verbatim. Audio recordings were deleted upon completion of transcription. The analytic approach was adapted to the objectives of each phase of the Double Diamond process, following established procedures for qualitative content analysis [26, 27].

In the *Discover* phase, semi-structured interviews with FCs were analysed inductively, allowing themes to emerge directly from the data without imposing preconceived frameworks. In the *Define* phase, focus group discussions were examined deductively, using the thematic framework generated in the *Discover* phase to validate, clarify, and expand the identified themes. In the *Deliver* phase, focus group feedback on the final programme was again analysed inductively to capture novel insights, unanticipated suggestions, and potential refinements.

Across all phases, two researchers (MPin and ASR) independently coded the transcripts, compared interpretations, and resolved discrepancies through iterative team meetings to ensure interpretive consensus and thematic saturation. This process fostered a high degree of agreement and established a shared interpretation of the data. The combined inductive–deductive approach ensured that findings remained firmly grounded in participants’ lived experiences while being progressively organised, verified, and refined into a coherent and comprehensive framework.

### Ethical approval

The study was approved by the National Ethical Committee (Comité de Protection des Personnes Est II; Ref. No. SI 22.00264.000072 / National No. 2021-A00553-38) and registered under the national clinical trial number NCT05651555. All participants read and signed a written informed consent form covering both participation in the study and the publication of their anonymised case descriptions. They were informed that all descriptions would remain strictly anonymous. The study was conducted in accordance with the ethical principles outlined in the Declaration of Helsinki.

## Results

### Participant characteristics and distribution across co-design phases

A total of 62 individuals —45 FCs and 17 healthcare professionals— participated in the study.

The 45 FCs (37 women, 8 men) had a mean age of 64.7 years (SD = 11.2; range: early 40s to late 80s). Their level of education was the following: No schooling *n* = 5, Primary *n* = 7, Secondary *n* = 8, Tertiary *n* = 25.

The 17 professionals (13 women, 4 men) had a mean age of 46.3 years (SD = 9.5; range: early 30s to late 60s) and a professional experience in geriatrics ranging from 5 to 25 years.

The detailed characteristics of both FCs and professionals, including the relationship of FCs to the person with dementia, the professional roles represented, and the specific actions in which participants were involved during each phase of the Double Diamond co-design process are presented in Table 2 and in Additional file 4.

The following sections present the findings for each phase of the co-design process, beginning with the themes identified during the *Discover* phase.

**Table 2:** Participant characteristics and involvement in actions across the four phases of the double diamond co-design process

Phases	Actions and description	Participants characteristics
Discover	Individual interviews with FCs: *n* = 14 Mean duration: 75 min (range 55–110)	Gender: 11 F, 3 M Profile: wives (*n* = 7), husbands (*n* = 2), daughters (*n* = 4), son (*n* = 1)
Define	FG with FCs : *n* = 3; group sizes = 6, 6, 7 participants respectively Duration: 90 min	Gender: 16 F, 3 M Profile: wives (*n* = 13), husbands (*n* = 3 ), daughters (*n* = 3)
	FG with professionals: *n* = 2; group sizes: 5 and 6 participants respectively Duration: 90 min	Gender: 9 F, 2 M Profile: physicians (*n* = 2), psychologists (*n* = 7), nurses (*n* = 2)
Develop	Workshops with the co-design team (*n* = 8 in each workshop ) and FCs (*n* = 4) Duration: 120 min	Professionals gender 6 : F, 2 M Professionals profile: Physicians (*n* = 4), psychologists (*n* = 3), research scientist (*n* = 1) FCs gender: 4 F FCs Profile : wives (*n* = 2), daughters (*n* = 2)
Deliver	Programme sessions with FCs;, group size (*n* = 8 ) and professionals; group size (*n* = 6) Duration: 90 min FG with FCs : *n* = 1; group size = 8 FG with professionals: *n* = 1; group size = 6	FCs gender: 6 F, 2 M FCs Profile: wives (*n* = 4), husband (*n* = 1), daughters (*n* = 3) Professionals gender: 4 F, 2 M Professionals profile: physicians (*n* = 2), psychologists (*n* = 2), nurses (*n* = 2)

*FCs *Family Caregivers,* FG *Focus groups,* F *Female* M *Male

### Discover phase: needs assessment through qualitative exploration

The qualitative needs assessment identified ten interrelated thematic domains reflecting the emotional, relational, and structural challenges faced by FCs during and after the transition of a relative with dementia into institutional care. These themes emerged from 14 semi-structured interviews with FCs. The following subsections describe each theme, illustrating them with representative participant quotations.

#### Health journey and admission to the institution

Many FCs described a long and emotionally taxing health journey that preceded their relative’s admission to an institutional long-term care facility. This journey often involved a gradual but undeniable decline in physical and cognitive abilities, such as recurrent falls, episodes of confusion, memory loss, and, in some cases, behavioural disturbances, that made it increasingly difficult to maintain care at home. Repeated hospitalisations, escalating care needs, and caregiver exhaustion, signalled the limits of home support systems, despite the presence of aides or regular medical assistance.

*“We tried everything at home. But after the second fall and another hospital stay*,* we knew we couldn’t do it anymore.”* (FC, daughter).

The decision to place a loved one in a facility was described as either a slow, deliberate process or a sudden, crisis-driven one. In both situations, FCs frequently experienced inner conflict: while the decision brought relief from physical and emotional exhaustion, it also evoked deep feelings of guilt, sadness, and, for some, a sense of betrayal.

*“It was either put her in or lose myself completely. But still*,* I feel like I betrayed her.”* (FC, husband).

This emotional ambivalence was further intensified when the relative either refused or reluctantly accepted institutionalisation, and when FCs felt pressured by professionals or isolated from other family members during the decision-making process.

*“My mother didn’t want to go… I feel like I stole her decision.”* (FC, daughter).

#### Caregiver burden at home

Before institutionalisation, many FCs described carrying an immense physical, emotional, and administrative load. They were responsible not only for daily personal care but also for coordinating medical appointments, managing paperwork, liaising with healthcare providers, and ensuring that the home environment remained safe and adapted to their relative’s evolving needs.

*“I was up twice a night for months. You stop being a daughter and become a nurse*,* a secretary*,* everything.”* (FC, daughter)

This constant vigilance often came at a significant personal cost, including chronic fatigue, disrupted sleep, heightened stress, and emotional strain.

*“I started having panic attacks*,* and even then I didn’t stop. You just keep going because there’s no other choice.”* (FC, wife)

Many FCs reported having little or no support from siblings or extended family, which contributed to feelings of isolation, injustice, or resentment.

“My brothers were far away… everything fell on me.” (FC, daughter)

For some, the prolonged strain resulted in a noticeable decline in their own physical and mental health, compounded by the loss of social interactions, reduced ability to maintain paid employment, and the erosion of personal wellbeing.

#### Family caregiver role after institutionalisation

Even after their relative entered an institutional long-term care facility, the caregiving role did not end—it simply changed in nature. FCs continued to visit frequently, monitor the quality of care, personalise the resident’s environment, and attend to their emotional wellbeing.

*“I went every day. I checked to make sure he was well cared for.”* (FC, wife).

While the physical demands of caregiving were reduced, many expressed a strong need to remain actively involved—both out of affection and from concern that care might otherwise become impersonal or insufficient. Several FCs described difficulties in finding a new equilibrium between presence and distance, balancing trust in staff with the desire to maintain oversight and decision-making power. In some cases, differences in expectations between FCs and staff created tension or required negotiation.

For others, the transition brought partial relief and an opportunity to reclaim aspects of their identity beyond caregiving, even if only partially.

*“It was a relief in some ways… I could finally sleep through the night*,* but I’m still her daughter*,* still her carer in my own way.”* (FC, daughter).

#### Relationship with the institution

FCs’ experiences with institutional long-term care facilities varied widely, but communication consistently emerged as a central concern. Many emphasised the importance of having reliable, empathetic, and accessible staff with whom they could speak regularly. Trust in the institution was closely tied to the quality, frequency, and openness of these interactions.

While some FCs described positive relationships with individual nurses or aides, others pointed to systemic shortcomings such as lack of transparency, unclear allocation of responsibilities, delayed responses to concerns, or occasional care errors.

*“No one explained what was going on*,* and I felt like I was in the way of the staff doing their work.”* (FC, husband).

There was also a strong desire for more personalised care, respecting the resident’s life history, routines, and preferences, as well as for activity programmes that addressed social, emotional, and cognitive needs beyond basic medical care.

*“It’s not just a room*,* it’s their home now. You have to know their story.”* (FC, wife).

Across these accounts, FCs saw themselves as essential partners in upholding the dignity and quality of life of their loved ones, advocating for their needs, and ensuring that care was truly person-centred. In this way, caregivers viewed themselves as essential partners in upholding the dignity of their loved ones.

#### Emotional and psychological impact

The emotional impact of caregiving, and particularly the process of placing a loved one in an institution, was described as profound. FCs reported feelings of guilt, grief, and inner conflict, especially when the decision to institutionalise was made under duress, in the context of a crisis, or when it was perceived as stigmatising by other family members or the community.

*“I didn’t know who I was without her at home.”* (FC, husband).

Some described the experience as a form of “living grief” or anticipatory mourning, as their loved ones gradually changed or lost aspects of their personality due to the progression of dementia. Others spoke of persistent anxiety, hypervigilance, or an enduring sense of failure despite their best efforts to provide care.

*“Even though he’s there now*,* I can’t let go—I keep checking everything*,* every day. It’s like my body rests*,* but my mind doesn’t.”* (FC, wife).

For a smaller number, however, the experience offered an opportunity for personal growth, reflection, and resilience, prompting them to find meaning and renewed purpose in their caregiving journey.

*“It forced me to step back and think about what really matters—for her and for me. I’ve learned to be present in a different way.”* (FC, daughter).

#### Family relationships and personal history

Family dynamics often played a central role in shaping how caregiving was experienced. Many FCs spoke of tensions with siblings or absent relatives, particularly when responsibilities were unequally distributed. Judgement, lack of involvement, or unresolved family conflicts often resurfaced during the caregiving period, compounding the emotional burden.

Some caregivers described personal histories marked by parental favouritism, unspoken promises, or past trauma, all of which influenced their sense of duty, loyalty, or frustration in the role.

*“I made a promise to my father. And my sisters resented me for it.”* (FC, daughter).

For some, being the primary caregiver also brought a sense of pride or moral obligation, particularly when reinforced by cultural values, generational expectations, or established family roles.

*“They always told me I was ‘the responsible one’. So it naturally fell to me.”* (FC, daughter of a couple).

#### Spirituality, values, and rituals

For several FCs, spirituality provided an important framework for understanding their caregiving role and finding solace during the transition to institutional care. Religious practices, such as attending mass, praying, or receiving communion, were described as essential touchpoints, supporting not only the resident’s sense of continuity but also the caregiver’s own emotional resilience.

*“Mass*,* prayer… it helped me keep going.”* (FC, wife).

The absence of rituals surrounding death, loss, or grief in the institutional setting was often experienced as a painful gap. Some FCs expressed a wish to integrate spiritual practices into the care environment or to honour their loved one’s life story through personalised ceremonies. These accounts highlight the role of meaning-making and ritual as sources of comfort and connection in the face of progressive illness and eventual loss.

*“I know the end will come*,* but here there’s nothing to prepare us—no space*,* no ritual. I’d like something that helps us face it together.”* (FC, wife).

#### Resources, support, and assistance

The availability and quality of support networks greatly shaped FCs’ caregiving experiences. Some were able to rely on family members, friends, or professional contacts, while others felt entirely alone in their responsibilities.

*“My daughter was there*,* she helped me a lot. Thankfully.”* (FC, wife).

Many emphasised the importance of emotional validation and broader societal recognition of their role, which they felt was often invisible or undervalued. Support groups, therapeutic spaces, and community-based programmes, particularly those that connected FCs with others in similar situations, were frequently described as vital resources. Such spaces not only provided practical advice but also reduced feelings of isolation and helped caregivers feel understood.

*“Being able to talk to others going through the same thing… it changes everything.”* (FC, husband).

#### Coping strategies and personal resources

FCs described developing a variety of strategies to manage stress and maintain balance in their daily lives. These included engaging in restorative activities such as walking in nature, reading, or pursuing spiritual practices. Some also chose to limit the frequency of their visits to preserve their mental health.

*“Walking in nature clears my head.”* (FC, daughter).

Others practised meditation or relaxation techniques, focused on family projects, planned travel, or made future plans that helped them reconnect with a sense of self beyond caregiving. Despite the diversity of approaches, many FCs expressed a common desire to safeguard their health, reclaim their identity, and intentionally make space for personal wellbeing.

#### Ethical, social, and existential reflections

For many participants, caregiving prompted deeper ethical and philosophical questioning.

*“Is it really living when they don’t even recognise you anymore? But then who am I to decide that?”* (FC, daughter).

Some FCs wrestled with concerns about medical overreach or “futile care,” questioning the value of interventions that prolonged life without quality. Others reflected on death, the fragility of memory, and their own fears of future cognitive decline.

*“It made me reflect on my own old age*,* on what I want for myself.”* (FC, daughter).

The experience of institutionalisation also sparked critical reflections on how society approaches ageing and vulnerability. In some cases, this awareness led FCs to engage in civic or spiritual initiatives, document their experiences, or advocate for more humane and person-centred care models. Through these reflections, the role of FC became a lens for examining broader existential, ethical, and social concerns.

### Define phase – thematic analysis and stakeholder validation

The focus groups with FCs and professionals built directly on the findings of the *Discover* phase, using the interview-derived themes as a starting point but allowing new, collective perspectives to reshape and expand them. This stage served a dual purpose: first, to validate and deepen the understanding of the issues previously identified, and second, to identify gaps, priorities, and practical suggestions for intervention that were less prominent, or absent, in individual interviews. While some topics from the initial framework were retained, others were reorganised or merged to better reflect the shared concerns emerging from group discussions. The results below present the defined thematic structure, integrating both confirmed insights and newly identified needs that informed the co-construction of the psychoeducational programme.

#### Ongoing burden after institutionalisation

Both FCs and professionals emphasised that the burden of care does not end with institutionalisation, it simply changes form. While physical tasks may decrease, emotional and logistical responsibilities continue. FCs described a sense of dispossession, particularly over hands-on tasks such as medication management or hygiene, which had previously defined their caregiving identity. This shift often brought feelings of guilt, powerlessness, and concern that care might become impersonal in the hands of overstretched staff.

*“I feel like I’m abandoning them*,* even though I’m here every day.”* (FC, daughter).

Professionals confirmed that families often arrive at the institution already emotionally and physically exhausted after a prolonged period of decline, crisis, or burnout.

*“When families arrive*,* they are often already emotionally and physically depleted*,* having endured years of decline*,* repeated crises*,* and constant vigilance.”* (Physician).

#### Communication challenges

Communication between FCs and institutions emerged as a central and recurring issue. Relationships with staff ranged from warm and supportive to distant and opaque. A frequent frustration was not knowing who to approach or how to raise concerns.

*“We never really know who to talk to*,* or how.”* (FC, daughter).

Professionals validated these concerns, citing systemic barriers such as lack of time, staff turnover, and insufficient training in family-centred communication. Both groups recognised the need for clearer and more empathetic communication tools to support constructive collaboration.

#### Emotional impact of transition

The transition from home to institutional care was described as emotionally taxing and morally burdensome. FCs spoke of guilt, anticipatory grief, and loneliness in decision-making, alongside feelings of exclusion from their loved one’s daily life once institutionalised.

*“They don’t know everything we still do.”* (FC, wife).

Professionals reported encountering high levels of caregiver distress, which could manifest as anxiety or even verbal aggression, often signs of deeper suffering and unacknowledged needs for support.

*“When they get upset with us*,* it’s often not anger—it’s pain. They feel powerless and unheard.”* (Psychologist).

#### Redefining the FC role

FCs described themselves as continuing to play an active role in their relative’s care: visiting regularly, personalising rooms, advocating for individualised attention, and monitoring quality of care. However, they noted that this ongoing contribution was not always acknowledged by staff.

*“I’m still very involved. I come often*,* I know exactly what reassures him*,* what upsets him*,* what he needs—but sometimes I feel like that knowledge isn’t really taken into account.”* (FC, daughter).

Professionals recognised this oversight and expressed willingness to improve relationships, though systemic constraints often limited their capacity.

*“We know families still have a lot to contribute*,* and we would like to involve them more. But with the workload and lack of time*,* it’s not always easy to create that space.” (Nurse).*

#### Coping strategies

Focus group discussions reinforced the coping strategies described in interviews. FCs drew on nature, prayer, reading, and relaxation techniques to maintain balance. Some limited visit frequency to preserve mental health.

*I’ve learned to protect myself. Sometimes I come less often*,* I go for walks*,* I pray… otherwise I wouldn’t be able to cope.”* (FC, wife).

While these practices fostered resilience, they were usually developed in isolation. Few FCs reported receiving institutional support for self-care.

*“Families often find their own ways to cope*,* but we rarely have structured ways to support them in that process.” (Psychologist)*.

#### New gaps identified

Focus groups also revealed important unmet needs not fully captured in the interviews. One of the most prominent was the lack of accessible information and training for FCs. Many reported ongoing confusion about how the institution functions—its rules, structures, and key points of contact—even after months of involvement. Others felt underinformed about dementia itself, particularly regarding behavioural and cognitive changes, which left them unprepared to understand their loved one’s loss of autonomy or changes in personality:

*“I realised I knew very little about Alzheimer’s… Understanding the disease and why certain behaviours happen would certainly change the way I approach my mother.”* (FC, daughter).

Nutrition was another recurring concern, with caregivers voicing frustration over meal quality, the dining environment, and the lack of transparency about adapted textures and dietary needs.

Communication difficulties emerged as a cross-cutting challenge, particularly in interactions with the resident. As cognitive decline progressed, FCs found it increasingly difficult to maintain verbal and emotional connection. Many described this gradual disconnection as a form of “silent grief,” marked by the painful awareness that their loved one was physically present yet psychologically distant.

*“It’s like they’re still here*,* but you can’t reach them anymore.”* (FC, wife).

Professionals added further insights, noting frequent encounters with family members who were visibly distressed, overwhelmed, or unsure how to navigate the institutional system. The absence of advance care planning was seen as a major systemic gap, often leading to ambiguity and conflict during end-of-life decision-making:

*“They don’t feel comfortable bringing it up with their relatives*,* but this can become an issue when the relative is nearing the end of life.”* (Physician).

Staff also acknowledged their own emotional fatigue, expressing a genuine desire to support families but feeling constrained by workload, understaffing, and insufficient relational training.

*“We’d like to do more for families*,* but we just don’t have the means.”* (Nurse).

#### Support preferences and programme design recommendations

Focus group discussions revealed a shared need among FCs for safe, structured spaces to process emotions, share experiences, and seek guidance. Preferences varied: some wished for group sessions to connect with peers, exchange practical advice, and feel less isolated:

*“I wish someone had offered me a group*,* just to talk about it.”* (FC, wife).

Others preferred individual sessions with professionals, valuing the opportunity for personalised attention:

*“I wish I could have a meeting with a psychologist*,* just to focus entirely on my own situation.”* (FC, daughter).

Building on these preferences, both FCs and professionals recommended that the psychoeducational programme be concise and flexible, comprising 8 to 10 sessions of 60–90 min each, with 10 to 12 FC participants. Sessions would be facilitated by a rotating multidisciplinary team with expertise in dementia care and institutional management (e.g., physician, psychologist, head nurse, healthcare manager, or director), each addressing topics aligned with their professional background. While group sessions would form the programme’s core, individual meetings—particularly at the beginning and end—would help tailor support to each FC.

### Develop phase : structuration of the psychoeducational programme

The Develop phase focused on translating the needs and priorities identified in earlier phases into a coherent psychoeducational programme for FCs. Through collaborative workshops, members of the co-design team, FCs, and healthcare professionals worked together to ensure that the intervention addressed real-world challenges while remaining feasible within institutional care settings.

The process began with consolidating findings from the Discover and Define phases into a structured synthesis linking each caregiver challenge to a proposed solution (programme content), the skills needed to address it, and the future corresponding program session. This mapping, presented in Table 3, served as a blueprint for the programme structure.

**Table 3:** Caregiver challenges, corresponding programme components, and targeted skills for the intervention

Caregiver challenges	Proposed solution (programme content)	Skills to develop	Programme session
Unmet needs and dissatisfaction; High stress levels; Crisis situations; and adaptation difficulties	Support caregivers in identifying and expressing personal needs and priorities; provide brief training on recognising and managing stress	Communication for needs expression; coping with stress and emotions; adaptability and flexibility	Individual session 1: Caregiver history; One-to-one needs assessment and stress management planning.
Loss of control and caregiving role	Discussion on transition to institutional care and ways to remain meaningfully involved	Identity adjustment; role redefinition; collaborative engagement; advocacy	Group session 2: Getting to know the group and redefining the caregiver role after institutionalisation.
Feeling excluded from the resident’s daily life	Reframe presence as emotionally meaningful; identify new modes of involvement	Emotional connection; balanced engagement	
Limited knowledge of dementia	Psychoeducation on dementia progression, symptoms, behaviours, autonomy, and personality change	Disease knowledge; health literacy; reframing behaviours	Group Session 3: Improving dementia literacy
Concerns about nutrition	Session on meal routines, adapted textures, and decision-making processes	Understanding adapted care; acceptance of routines; emotional regulation	Group Session 4: Nutrition
Lack of knowledge about institutional functioning	Session on institutional organization: hierarchy, communication protocols, and key contacts	Navigating institutional systems; assertive communication; seeking clarification	Group session 5 : Understanding the institution functioning
Perceived insufficiency of care in the institution	Explanation of organisational roles, constraints, and institutional care delivery	Institutional literacy; realistic expectations	
FC’s emotional distress and aggressive behavior due to overload	Stress and emotion management strategies; optional individual psychological support	Stress and emotion management strategies; optional individual psychological support	Group session 6: Managing stress
Communication difficulties with the resident	Training in alternative communication strategies in advanced dementia and non-verbal tools	Empathic and adaptive communication, patience, non-verbal communication strategies	
Isolation and lack of support in decision-making and end-of-life topics	Group discussion on end-of-life topics and anticipatory directives; peer support, and expression of emotions	Decision-making under stress; boundary setting; emotional support seeking	Group session 7: Considering end of life
Poor communication with staff	Dedicated session on communicating with professionals; role-playing or scenarios; tools for constructive dialogue	Communication strategies; assertiveness; negotiation and emotional de-escalation; trust building	Group session 8: Communicating with professionals
Persistent unmet needs after programme: stress or emotional burden; uncertainty about applying learnings in daily life	Review and prioritise needs; plan next steps or referrals; reinforce coping strategies; suggest ongoing support options; create a personalised action plan with concrete examples	Self- assessment and problem-solving; emotional regulation and resilience; integration of skills into caregiving routines	Individual session 9: Programme review and follow-up planning

Building on this framework, the team developed a detailed session plan outlining titles, core content, learning objectives, and facilitators (Table 4). Each session was designed to address specific needs identified in earlier phases, integrate skill-building opportunities, and draw on multidisciplinary expertise.

**Table 4:** Titles, content, and objectives of the caregiver programme sessions in nursing homes

Session title	Content covered	Session Objectives	Facilitators
Session 1: Initial individual interview	- Participant history	- Assess the relationship between the family caregiver and the care recipient. - Identify the caregiver’s needs, sources of dissatisfaction, stressors, goals, and motivation for participating in the programme.	Psychologist
Session 2: Programme Introduction: goals and content	- Presentation of programme goals and content - Discussion of participants’ expectations	- Familiarize caregivers with the programme structure - Discussion of participants’ expectations - Sharing participants’ experiences and creating connections	Psychologist and geriatrician
Session 3: Memory-related and neurodegenerative diseases	- Overview of neurodegenerative diseases and associated behavioral disorders	Understand disease progression and behavioral impacts - Accept the prevalence of neurodegenerative conditions in nursing homes Recognize remaining capacities of their relative (beyond lost abilities) - Adapt behaviors to their relative’s current state - Acknowledge institutionalization as an appropriate solution for advanced stages	Psychologist and geriatrician
Session 4: Malnutrition, risks, and challenges	- Risks of malnutrition, choking hazards, and food refusal	- Explain nutritional risks in neurodegenerative diseases - Present practical solutions for dietary management	Psychologist and Geriatrician
Session 5: “A day in the life of a resident”	- Roles of staff in nursing homes - Typical daily routine of residents	- Understand institutional operations - Identify key staff contacts for specific needs - Align expectations with nursing homes realities - Obtain detailed health updates about their relative	Psychologist and health care manager
Session 6: Psychological challenges of institutionalisation	- Participants’ emotional experiences during their relative’s transition	- share Participants’ emotional experiences during their relative’s transition	Psychologist and healthcare manager
Session 7: End-of-life care	- Legal frameworks for end-of-life care - Advance directives and trusted third parties	- Discuss end-of-life support resources in nursing homes - Create a safe space to share fears/emotions - Replace avoidance of death with proactive planning	Psychologist, geriatrician and facility director
Session 8: programme Synthesis	- Participation history - Ongoing interrogations	- Evaluate the programme’s impact on caregivers - Identify tailored solutions for ongoing stressors	Psychologist and geriatrician
Session 9: Final individual interview	Synthesis of programme	- Review the programme with the caregiver. - Reinforce their knowledge. - Evaluate changes in dysfunctional patterns, - Explore new issues that may have emerged	Psychologist

The resulting psychoeducational programme was structured to be practical, flexible, and scalable. It comprised nine weekly sessions over nine weeks: two individual interviews (before and after the programme) and seven thematic group workshops, each lasting approximately 90 min. Sessions were co-facilitated by a rotating multidisciplinary team—including a geriatrician, facility director, and healthcare manager—while a psychologist ensured continuity throughout.

The initial individual interview explored the caregiver–care recipient relationship, identified needs, stressors, and goals, and addressed crisis situations and adaptation challenges. Group workshops combined short expert presentations, peer discussions, practical exercises, and multimedia resources, covering: 1) Programme Introduction: goals and content; (2) Memory-related and neurodegenerative Diseases; (3) nutrition in institutional care; (4) “A day in the life of a resident”: institutional structure and governance; (5) Psychological impact on caregivers; (6) end-of-life laws and support; and (7) Synthesis of key learnings and planning next steps. The final individual interview reviewed the caregiver’s experience, reinforced acquired knowledge, assessed changes in coping strategies, and addressed any outstanding or emerging concerns.

### Deliver phase: Implementation of the psychoeducational intervention and participant feedback

The first version of the psychoeducational intervention was pilot-tested with eight FCs and six professionals. FCs attended all seven thematic group workshops and two individual sessions, while professionals participated only in the group sessions. Feedback was collected at the end of each session and during two concluding focus groups—one with FCs and one with professionals—to evaluate the programme’s feasibility, acceptability, clarity, perceived benefits, and areas for improvement. The following sections present participant feedback organised into these main dimensions, followed by the professionals’ perspectives and the adjustments made to finalise the programme for feasibility testing.

#### Feasibility and acceptability - family caregivers perspectives

Participation in the programme was high, with no dropouts among FCs. The number of sessions, their one-hour duration, and the location within a geriatric facility were considered appropriate and manageable. The combination of group and individual sessions was seen as well-balanced—group sessions offered opportunities to share knowledge and experiences, while individual consultations allowed for personalised guidance. During the group sessions, FCs appreciated the combination of expert presentations and open discussion:

*“The number of sessions was right*,* the location in a geriatric institution was convenient*,* and the group and individual formats worked really well.” (FC*,* spouse)*.

*“The workshops offer a different view of the institution and the professionals who work there.” (FC*,* daughter)*.

FCs confirmed that the structure fit their needs and highlighted the openness of exchanges and the respectful atmosphere. They also praised the professionalism and empathy of the facilitators:

*“The team was very dynamic and motivated*,* and approachable for advice if needed.” (FC*,* daughter)*.

*“Congratulations! I hope this training will continue to support many caregivers who are going through great difficulties.” (FC*,* husband)*.

#### Clarity of content

Both FCs and professionals found the programme’s objectives and content clear, well-structured, and accessible. Presentations were generally described as easy to follow and appropriately paced:

*“The explanations were understandable and clear.” (FC*,* spouse)*.

The use of plain language and concrete examples was appreciated, helping participants grasp complex topics such as dementia progression, institutional procedures, and care planning. Visual aids and the alternation between expert input and discussion were also seen as helpful in maintaining engagement.

However, certain topics—particularly the end-of-life care module—were described as emotionally challenging, especially when sensitive clinical details were presented early in the session:

*“The palliative care part was a bit emotionally sensitive.” (FC*,* daughter)*.

To reduce distress and allow participants to process the information more gradually, several suggested reorganising this session so that administrative and legal aspects (e.g., advance directives, decision-making processes) are presented first, followed by clinical and emotional issues. This sequencing was seen as a way to create a more supportive environment for discussing sensitive matters.

#### Key benefits for family caregivers

Participation in the programme brought substantial and multifaceted benefits for FCs, combining practical knowledge, emotional relief, and a renewed sense of their role within the institutional setting. A frequently mentioned gain was a better understanding of how geriatric institutions function, their organisation, the roles of different professionals, and the constraints under which they operate. For some, this transformed a previously opaque system into something more approachable:

*“After six months*,* I still didn’t know how it [the institution] worked (laughs)*,* who did what…*,* it was a mystery*,* really a screen.” (FC*,* daughter)*.

*“The workshops offer a different view of the institution and the professionals who work there.” (FC*,* daughter)*.

This knowledge increased their confidence in addressing concerns and communicating effectively with the appropriate personnel, while also fostering empathy and mutual understanding with staff:

*“It helps to think differently about what we face*,* and to understand the difficulties on the institution’s side—organization*,* staffing*,* etc.” (FC*,* husband)*.

*“It leads us to trust the staff more—and when you know them*,* that trust is even stronger.” (FC*,* wife)*.

Many FCs also reported feeling better equipped to play an active and constructive role in the care environment. By better understanding institutional routines and constraints, they recognised opportunities to contribute in ways that eased staff workload without overstepping boundaries.

*“For many small things*,* we can actually relieve the staff of certain tasks (…) and be helpful.” (FC*,* wife)*.

The programme helped FCs recalibrate expectations and adjust their involvement to the institutional context, which strengthened their sense of competence and usefulness:

*“It helped me take a role that was more suitable*,* more appropriate than what I had imagined at first.” (FC*,* wife)*.

*“Gradually*,* I was able to take a somewhat useful place.” (FC*,* husband)*.

*“To be able to help to the point where you feel useful.” (FC*,* wife)*.

Beyond these practical aspects, participants valued the emotional relief gained from being able to share their experiences, doubts, and feelings of guilt in a safe, non-judgmental space:

*“During the workshops*,* I was able to talk about things I wouldn’t have otherwise.” (FC*,* daughter)*.

*“We share our own doubts and feelings.” (FC*,* wife)*.

Group sessions fostered solidarity and emotional validation, helping FCs to recognise that they were not alone in facing these challenges:

*“The discussions between participants were very positive.” (FC*,* son)*.

*“It may be the first time I’ve had real conversations on these subjects with people who also have a parent in a nursing home.” (FC*,* wife)*.

*“Being able to share our experiences was really valuable.” (FC*,* daughter)*.

For many, the programme improved their understanding of dementia progression and behavioural issues, leading to less stressful, more empathetic interactions with their loved ones:

*“Now I visit my mother more relaxed*,* and I have a pleasant time with her.” (FC*,* daughter)*.

Similarly, discussions on end-of-life planning helped participants adapt expectations and prepare for important decisions:

*“It helps prepare*,* reflect*,* and move forward with necessary decisions.” (FC*,* husband)*.

A final element frequently highlighted as essential to the programme’s impact was its combination of individual and group formats. The individual sessions, held at the beginning and end, provided a confidential space to discuss personal difficulties and receive tailored guidance:

*“Having time alone with the facilitator before and after the programme helped me focus on my own priorities and see the changes in how I handle things.” (FC*,* son)*.

*“In the individual sessions*,* I could express my doubts and get tailored advice—it really complemented the group work.” (FC*,* spouse)*.

#### Suggestions for improvement

Suggestions for improvement focused on enhancing preparation, extending the programme’s reach, and offering ongoing support. Several FCs expressed the wish to receive a brief outline of each session beforehand, allowing them to prepare specific questions and engage more deeply with the topics discussed:

*“Provide us with an outline of each session in advance so we can prepare and ask more specific questions.” (FC*,* husband)*.

Others proposed the creation of follow-up sessions, described as a “Level 2” of the programme, so that participants could revisit concepts, share progress, and address new challenges arising over time. There was also a strong call to widen access to the intervention, not only for FCs of residents already in care facilities but also for those supporting relatives still living at home, as this knowledge could facilitate a smoother transition when institutionalisation becomes necessary:

*“Make the programme known to a wider audience.” (FC*,* daughter)*.

*“This should ideally be discovered before the loved one enters a care facility*,* to help understand how things work and what everyone’s role is.” (FC*,* daughter)*.

#### Professional perspectives

Professionals echoed many of the FCs’ impressions, noting that the programme deepened caregivers’ understanding of institutional care and encouraged more collaborative attitudes toward staff:

*“Caregivers who were very anxious became less concerned about every detail of their loved one’s daily care.” (Professional*,* nurse)*.

They also noted a noticeable shift in FCs’ attitudes over the course of the programme. As participants gained a better understanding of institutional routines and constraints, they became less inclined to intervene in day-to-day care decisions and more willing to trust staff judgment. This shift, in turn, contributed to more constructive and cooperative relationships between FCs and professionals:

*“Some caregivers would tell others*,* ‘Let it go*,* you’re only hurting yourself…’” (Professional*,* psychologist)*.

Professionals reported gaining valuable insights into FCs’ emotional challenges and expectations, which enhanced their capacity to engage with families more empathetically. They proposed structural improvements, such as using short videos to ensure key content is delivered even when sessions are disrupted by questions, and reorganising emotionally sensitive sessions like those on palliative care:

*“It would be useful to have a short film at the beginning to summarise the main messages.” (Professional*,* geriatrician)*.

*“Starting with the administrative part makes it easier before moving on to palliative care—it’s less overwhelming that way.” (Professional*,* geriatrician)*.

#### Finalization and preparation for feasibility testing

The final stage of the development process focused on consolidating and formalising the psychoeducational intervention to ensure it was ready for wider implementation. Drawing on the feedback gathered during the *Deliver* phase, the co-design team systematically reviewed each component to maximise its relevance, coherence, and practical applicability in geriatric care settings.

Several targeted refinements were made. Brief summary videos were added at the start of each group session to reinforce the main messages and provide a consistent baseline of information, even if discussions diverged. Emotionally sensitive modules—such as those on palliative and end-of-life care—were reorganised to introduce administrative and legal aspects first, before addressing clinical and emotional issues, thereby easing participants into potentially distressing topics. The final session was strengthened with practical communication tools and role-play exercises to help FCs engage more effectively with institutional staff.

To ensure uniform delivery across sites and facilitators, a comprehensive facilitator manual was produced. This included session-by-session learning objectives, detailed content outlines, discussion prompts, and timing recommendations. The manual also specified the required audiovisual equipment, printed materials, and room setup to optimise participation.

In parallel, the research team developed the methodological framework for the upcoming feasibility study, including eligibility criteria, recruitment strategies, and outcome measures.

## Discussion

This study expands the evidence base on family caregiving in the context of institutionalisation, providing detailed insights into the psychoeducational needs of FCs and describing the collaborative development of a targeted intervention. Using a participatory co-design methodology that actively engaged both FCs and healthcare professionals, the research addresses long-standing calls for more inclusive and contextually grounded approaches in geriatric care [28, 29]. Consistent with Muller et al. [17] recommendations for the staged development of complex interventions, the work integrated iterative stakeholder feedback and pilot testing to assess feasibility and acceptability before progressing to broader implementation.

The analysis highlights four interrelated domains where caregivers benefit most from structured support: redefining their caregiving role after institutionalisation, improving communication with professionals, increasing knowledge of dementia and care systems, and adjusting emotionally to the transition. These findings are discussed below in relation to existing literature, with particular attention to how the Double Diamond model shaped both process and outcomes.

### Value of a participatory co-design approach for programme development

The Double Diamond model [22, 23] provided a structured yet adaptive framework for navigating the co-design process. Its alternating phases of divergent exploration and convergent synthesis enabled the systematic identification of diverse caregiver needs before refining them into actionable, feasible programme components. A central design feature, the mapping of specific caregiver challenges to proposed solutions and skills, translated abstract emotional and relational difficulties into practical learning objectives, aligning with Lazarus and Folkman’s [19] stress and coping theory.

Both FCs and professionals reported valuing their involvement in the programme’s conception, describing the process as a meaningful opportunity to have their perspectives directly shape the intervention. This finding is consistent with previous work showing that involving patients and caregivers in the design of health education and support programmes can enhance their acceptability and contextual relevance [22, 25].

Grounding the design in both lived experience and professional expertise enhanced the programme’s relevance and practicality, which is expected to improve its credibility and the likelihood of successful implementation in real-world settings.

### Role redefinition

In line with previous research [5, 9, 30–32], FCs in this study did not view institutionalisation as the end of their caregiving responsibilities but as a transformation of their role. They reported continued emotional investment, ongoing monitoring of care, and ambivalence about their place within the care relationship. The psychoeducational intervention directly addressed this transition, helping caregivers to construct a new identity grounded in emotional presence, advocacy, and continuity of the relationship. Drawing on their intimate knowledge of the resident’s preferences and needs, and being supported to act as advocates for the best quality of care possible, may enable FCs to feel integrated into the ‘specialist’ care team, as suggested by Camoes-Costa et al. [33]. By reframing their role, FCs are able to find a more balanced and sustainable form of engagement.

### Communication with professionals

One of the most significant contributions of this work is its attention to relational dynamics between families and professionals. Communication breakdowns and relational asymmetries remain persistent barriers in institutional care [5–9, 18, 34, 35]. Our findings confirm that FCs often feel marginalised, excluded, or misunderstood by professional staff, experiences that can exacerbate emotional distress and lead to conflict. By incorporating role-specific communication tools and structured sessions on navigating institutional systems, the psychoeducational programme aimed at restoring trust and fostering constructive dialogue. Feedback indicated that these sessions were highly valued, with participants suggesting the addition of practical tools, such as role-playing exercises and communication scripts, to further strengthen applied skills.

### Education on dementia and institutional care systems

The inclusion of targeted educational modules on Alzheimer’s disease, nutrition, and end-of-life care was particularly impactful, echoing previous findings [6, 9, 36]. Structured information on disease progression and behavioural changes directly addressed the communication and knowledge gaps identified in the thematic analysis. As suggested by previous authors [6, 9, 14–16], by deepening their understanding of dementia, FCs were better able to interpret patient behaviours, respond appropriately, and maintain more satisfying and less emotionally taxing relationships. Nutrition-focused sessions—on swallowing difficulties, texture adaptation, and mealtime routines—reframed feeding challenges as adapted care rather than neglect, countering frustrations voiced in focus groups. End-of-life care modules created a safe space to discuss advance directives, ethical dilemmas, and emotional preparedness, responding to the avoidance of these topics noted by both FCs and professionals, as recommended by previous authors [14, 37].

Education on institutional care systems—covering organizational structures, staff roles, communication pathways, and procedural norms—was equally critical, as mentioned in previous works [16, 33, 36]. This content reduced feelings of exclusion, clarified points of contact, and fostered more constructive collaboration with staff. By providing a better understanding of how the facility operated, FCs felt reassured, empowered and were more likely able to answer their relative’s questions, and accompany them in the transition from home to a nursing home.

### Emotional adjustment and programme format

Emotional adjustment emerged as a core need throughout the process of institutionalisation, as emphasized by previous authors [5, 9, 16, 36]. The blended format—seven thematic group sessions complemented by two individual consultations—proved particularly effective in addressing this need. The initial individual session enabled facilitators to assess each FC’s situation, identify sources of distress, and set personalised objectives. Group sessions fostered shared reflection, peer validation, and collective problem-solving, producing a normalising effect that reduced isolation. The final individual consultation consolidated learning, evaluated changes in emotional well-being, and identified strategies for ongoing adaptation.

This format combined the intimacy and tailoring of one-to-one work with the social support and perspective of peer groups, differing from previous approaches limited to either individual [11–14, 16] or group [15, 19] interventions. In the present study, FCs described the group experience as transformative, citing increased resilience, a renewed sense of normality, and improved capacity to manage the ongoing challenges of dementia care, echoing previous works [36, 37]. Individual sessions also allowed exploration of sensitive issues, work on specific goals, and adaptation of tools to each FC’s needs, while offering a confidential space for difficult emotions—further enhancing the programme’s overall impact.

### Policy implications

The study also raises important implications for policy and practice. Institutional systems often operate under time and staffing constraints that limit relational work. However, this research highlights that supporting caregivers is not an ancillary task, but a core component of person-centered dementia care. Embedding psychoeducational interventions within institutional routines—using brief, scalable formats and mixed delivery (in-person and video)—offers a feasible way to bridge the gap between families and care teams.

### Limitations

Despite its strengths, the study has limitations. The sample was limited to a specific geographic area (Île-de-France) and may not reflect the diversity of caregiver experiences across cultural or institutional contexts. Additionally, while the pilot phase of the intervention received positive feedback, long-term outcomes such as stress reduction, quality of life, and caregiver–health professional collaboration have yet to be evaluated. Future research should include longitudinal follow-up and comparison with control groups to assess the programme’s effectiveness over time.

## Conclusion

This study presents a brief, scalable psychoeducational intervention, co-designed with FCs and healthcare professionals using the Double Diamond model, to support families during the transition of a relative with dementia to institutional care. Grounded in lived experience and clinical insight, the programme addresses role redefinition, communication with professionals, and education on dementia and institutional systems, while fostering emotional adjustment through a blend of individual and group sessions. By embedding such structured, context-sensitive support within institutional routines, the intervention has the potential to strengthen family–professional partnerships, improve caregiver well-being, and enhance the relational quality of dementia care.

## Supplementary Information

Supplementary Material 1.

Supplementary Material 2.

Supplementary Material 3.

Supplementary Material 4.

## Data Availability

The data supporting our study are available on request.

## Abbreviations

FCs: Family Caregivers
FG: Focus groups
F: Female
M: Male

## References

[1] Groenvynck L, de Boer B, Beaulen A, et al. The paradoxical experiences of informal caregivers during the transition from home to nursing home. Innov Aging. 2022;6(Suppl 1):23. 10.1093/geroni/igac059.085.

[2] Gaugler JE, Mittelman MS, Hepburn K, Newcomer R. Clinically significant changes in burden and depression among dementia caregivers following nursing home admission. BMC Med. 2010;8(1):85. 10.1186/1741-7015-8-85. 21167022 10.1186/1741-7015-8-85 PMC3012012

[3] Afram B, Verbeek H, Bleijlevens MHC, Hamers JPH. Needs of informal caregivers during transition from home towards institutional care in dementia: a systematic review of qualitative studies. Int Psychogeriatr. 2015;27(6):891–902. 10.1017/S1041610214002154. 25287064 10.1017/S1041610214002154

[4] Fernandes CS, Angelo M. Family caregivers: what do they need? An integrative review. Rev Esc Enferm USP. 2016;50(4):675–82. 10.1590/S0080-623420160000500019. 27680055 10.1590/S0080-623420160000500019

[5] Lee K, Chung J, Meyer KN, Dionne-Odom JN. Unmet needs and health-related quality of life of dementia family caregivers transitioning from home to long-term care: a scoping review. Geriatr Nurs. 2022;43:254–64. 10.1016/j.gerinurse.2021.12.005. 34953331 10.1016/j.gerinurse.2021.12.005

[6] Harper AE, Terhorst L, Moscirella M, Turner RL, Piersol CV, Leland NE. The experiences, priorities, and perceptions of informal caregivers of people with dementia in nursing homes: a scoping review. Dementia. 2021;20(8):2746–65. 10.1177/14713012211012606. 33899537 10.1177/14713012211012606

[7] Givens JL, Lopez RP, Mazor KM, Mitchell SL. Sources of stress for family members of nursing home residents with advanced dementia. Alzheimer Dis Assoc Disord. 2012;26(3):254–9. 10.1097/WAD.0b013e31823899e4. 22037596 10.1097/WAD.0b013e31823899e4 PMC3288670

[8] Hui Z, Yang C, Fan Lee DT. Stressors and coping strategies in Chinese family caregivers of people with dementia in long-term care facilities: a qualitative descriptive study. Dementia. 2022;21(3):957–71. 10.1177/14713012211066661. 35130752 10.1177/14713012211066661

[9] Thompson G, Hack T, Rodger K, St. John P, Chochinov H, McClement S. Clarifying the information and support needs of family caregivers of nursing home residents with advancing dementia. Dementia. 2021;20(4):1250–69. 10.1177/1471301220927617. 32460548 10.1177/1471301220927617

[10] Ducharme F, Lévesque L, Lachance L, Giroux F, Legault A, Préville M. Taking care of myself: efficacy of an intervention programme for caregivers of a relative with dementia living in a long-term care setting. Dementia. 2005;4(1):23–47. 10.1177/1471301205049189.

[11] Gaugler JE, Roth DL, Haley WE, Mittelman MS. Can counseling and support reduce Alzheimer’s caregivers’ burden and depressive symptoms during the transition to institutionalization? Results from the NYU caregiver intervention study. J Am Geriatr Soc. 2008;56(3):421–8. 10.1111/j.1532-5415.2007.01593.x. 18179495 10.1111/j.1532-5415.2007.01593.x PMC2700042

[12] Gaugler JE, Birkeland RW, Albers EA, et al. Efficacy of the residential care transition module: a telehealth intervention for dementia family caregivers of relatives living in residential long-term care settings. Psychol Aging. 2024;39(5):565–77. 10.1037/pag0000820. 38753405 10.1037/pag0000820 PMC11552057

[13] Davis JD, Tremont G, Bishop DS, Fortinsky RH. A telephone-delivered psychosocial intervention improves dementia caregiver adjustment following nursing home placement. Int J Geriatr Psychiatry. 2011;26(4):380–7. 20842759 10.1002/gps.2537

[14] Schulz R, Rosen J, Klinger J, Musa D, Castle NG, Kane AL, Lustig A. Effects of a psychosocial intervention on caregivers of recently placed nursing home residents: a randomized controlled trial. Clin Gerontol. 2014;37(4):347–67. 25071302 10.1080/07317115.2014.907594 PMC4111253

[15] Paun O, Farran CJ, Fogg L, Loukissa D, Thomas PE, Hoyem R. A chronic grief intervention for dementia family caregivers in long-term care. West J Nurs Res. 2015;37(1):6–27. 10.1177/0193945914521040. 24510968 10.1177/0193945914521040 PMC5546622

[16] Zmora R, Statz TL, Birkeland RW, et al. Transitioning to long-term care: family caregiver experiences of dementia, communities, and counseling. J Aging Health. 2021;33(1–2):133–46. 10.1177/0898264320963588. 32990494 10.1177/0898264320963588 PMC7891851

[17] Müller C, Lautenschläger S, Meyer G, Stephan A. Interventions to support people with dementia and their caregivers during the transition from home care to nursing home care: a systematic review. Int J Nurs Stud. 2017;71:139–52. 28411508 10.1016/j.ijnurstu.2017.03.013

[18] Brooks D, Fielding E, Beattie E, Edwards H, Hines S. Effectiveness of psychosocial interventions on the psychological health and emotional well-being of family carers of people with dementia following residential care placement: a systematic review. JBI Database Syst Rev Implement Rep. 2018;16(5):1240–68. 10.11124/JBISRIR-2017-003634. 10.11124/JBISRIR-2017-003634 29762315

[19] Lazarus RS. Emotion and adaptation. New York: Oxford University Press; 1991.

[20] Slattery P, Saeri AK, Bragge P. Research co-design in health: a rapid overview of reviews. Health Res Policy Syst. 2020;18(1):17. 32046728 10.1186/s12961-020-0528-9 PMC7014755

[21] Szebeko D, Tan L. Co-designing for society. Australas Med J. 2010;3(9):580.

[22] Banbury A, Parkinson L, Gordon S, Wood D. Implementing a peer-support programme by group videoconferencing for isolated carers of people with dementia. J Telemed Telecare. 2019;25(9):572–7. 31631761 10.1177/1357633X19873793

[23] Banathy BH. Conversation in social systems design. Educ Technol. 1996;36(1):39–41.

[24] Design Council UK. The design process. Eleven lessons: managing design in eleven global brands. London: Design Council; 2005.

[25] Johnson M, Hutchinson A, Rattray J, McAuley D, Blackwood B, Andrews P. Co-design and evaluation of a digital family-led delirium prevention intervention for critically ill patients using the Double Diamond framework. J Crit Care. 2024;78:154291. 10.1016/j.jcrc.2024.154291.

[26] Elo S, Kyngäs H. The qualitative content analysis process. J Adv Nurs. 2008;62(1):107–15. 18352969 10.1111/j.1365-2648.2007.04569.x

[27] Fereday J, Muir-Cochrane E. Demonstrating rigor using thematic analysis: a hybrid approlder adultsch of inductive and deductive coding and theme development. Int J Qual Methods. 2006;5(1):80–92.

[28] Wicks PG, Reason P, Bradbury H. Living inquiry: personal, political and philosophical groundings for action research practice. The Sage handbook of action research: participative inquiry and practice. 2nd ed. London: Sage; 2008. pp. 15–30.

[29] Dorant E, Krieger T. Contextual exploration of a new family caregiver support concept for geriatric settings using a participatory health research strategy. Int J Environ Res Public Health. 2017;14(12):1467. 29182535 10.3390/ijerph14121467 PMC5750886

[30] Nolan M, Davies S, Brown J. Transitions in care homes: Towards relationship-centred care using the ‘Senses Framework’. Qual Ageing Older Adults. 2006;7(3):5–14.

[31] Gaugler JE, Mittelman MS, Hepburn K, Newcomer R. Predictors of change in caregiver burden and depressive symptoms following nursing home admission. Psychol Aging. 2009;24(2):385–96. 19485656 10.1037/a0016052 PMC2699253

[32] Hennings J, Froggatt K. The experiences of family caregivers of people with advanced dementia living in nursing homes, with a specific focus on spouses: a narrative literature review. Dementia. 2019;18(1):303–22. 10.1177/1471301216671418. 27856694 10.1177/1471301216671418

[33] Camões-Costa V, Loganathan J, Barton C, et al. Factors contributing to the mental health outcomes of carers during the transition of their family member to residential aged care: a systematic search and narrative review. BMC Geriatr. 2022;22(1):433. 10.1186/s12877-022-03105-4. 35581539 10.1186/s12877-022-03105-4 PMC9115935

[34] Hertzberg A, Ekman SL. We, not them and us? Views on the relationships and interactions between staff and relatives of older people permanently living in nursing homes. J Adv Nurs. 2000;31:614–22. 10718881 10.1046/j.1365-2648.2000.01317.x

[35] Zhao D, Shao H, Wang P, Xie L, Chen Z. Experiences of family caregivers and nursing home staff interactions during the adaptation process of elderly individuals moving to nursing home: a qualitative study. BMJ Open. 2024;14(10):e084138. 39395828 10.1136/bmjopen-2024-084138 PMC11474707

[36] Brooks D, Beattie E, Fielding E, Wyles K, Edwards H. Long-term care placement: the transitional support needs and preferences of spousal dementia caregivers. Dementia. 2022;21(3):794–809. 10.1177/14713012211056461. 34870490 10.1177/14713012211056461

[37] Gonella S, Mitchell G, Bavelaar L, Conti A, Vanalli M, Basso I, Cornally N. Interventions to support family caregivers of people with advanced dementia at the end of life in nursing homes: a mixed-methods systematic review. Palliat Med. 2022;36(2):268–91. 34965759 10.1177/02692163211066733

