# Supplementary material for: Co-designing a psychoeducational intervention for FCs of institutionalized older adults : a participatory double diamond approach
Source: BMC Geriatr. 2026 Apr 6;26:691. doi: 10.1186/s12877-026-07398-7 (PMC13188782; doi:10.1186/s12877-026-07398-7)
Supplement: Supplementary file 1 — Supplementary Material 1. [file 12877_2026_7398_MOESM1_ESM.pdf]

Additional File 1

"Discover phase" Interview Guide for family caregivers – Family Caregiver Needs and Motivation

Background and Context

- Can you tell me a little about your relationship with your relative?
- How long has your relative been in the geriatric institution?
- Could you describe the circumstances that led to the decision for institutionalisation?

Emotional Experiences

- How have you felt since your relative entered the institution?
- Can you describe moments that have been particularly difficult or positive for you during this period?
- Have your emotions changed over time since the admission? If so, how?

Caregiving Role Changes

- How has your role as a caregiver changed since your relative entered the institution?
- Which aspects of caregiving do you still carry out?
- Are there responsibilities you have found difficult to let go of or delegate?

Perceptions of Institutional Staff

- How would you describe your interactions with the staff?
- What has gone well in your communication with them?
- Have there been any challenges or misunderstandings?

Support Needs

- What types of support have you received since the institutionalisation?
- What kind of help or resources do you feel are missing?
- If you could design the ideal support programme for people in your situation, what would it include?
