# Supplementary material for: Co-designing a psychoeducational intervention for FCs of institutionalized older adults : a participatory double diamond approach
Source: BMC Geriatr. 2026 Apr 6;26:691. doi: 10.1186/s12877-026-07398-7 (PMC13188782; doi:10.1186/s12877-026-07398-7)
Supplement: Supplementary file 2 — Supplementary Material 2. [file 12877_2026_7398_MOESM2_ESM.pdf]

Additional File 2

"Define" Phase semi-Structured Focus Group guide

A. Family Caregivers

Introductions & Context

- Briefly introduce yourself and your relationship to your relative.
- How long has your relative been living in the institution?

Emotional Experiences

- How would you describe your feelings during the first weeks and months after your relative's admission?
- What were the most difficult emotional challenges you faced?

Changes in Caregiving Role

- How has your role changed since your relative entered the institution?
- What tasks or responsibilities do you still carry out?
- How do you feel about this new role?

Perceptions of Institutional Staff

- How would you describe your relationship and communication with staff?
- What has worked well? What could be improved?

Support Needs

- What types of information, resources, or emotional support would have helped you most during this transition?
- Were there moments when you felt particularly supported?
- Were there moments when you felt alone or excluded?

Recommendations for a Psychoeducational Programme

- What should it include?
- What topics would be most important to address?
- What format would work best for you (e.g., group, individual, online, in-person)?

B. Professionals

Introductions & Context

- Briefly describe your role and experience in working with families of newly admitted residents.

Observations on Family Caregiver Experiences

- From your perspective, what emotions and challenges do family caregivers face during the first months after admission?

- Are there common patterns or variations you have observed?

Caregiver Role Changes

- How do you perceive the change in family caregivers' roles after institutionalization?
- How do families adapt to this new role? Where do they struggle most?

Family–Staff Relationships

- How would you describe the communication and collaboration between families and staff?
- What helps build trust? What creates tensions?

Support Needs & Gaps

- What information or resources do you think families most need at the start of the institutionalization process?
- How well do current practices meet these needs?

Recommendations for a Psychoeducational Programme

- What content or skills should be prioritized to help families adapt?
- How can such a programme improve collaboration between families and professionals?
