# Supplementary material for: Co-designing a psychoeducational intervention for FCs of institutionalized older adults : a participatory double diamond approach
Source: BMC Geriatr. 2026 Apr 6;26:691. doi: 10.1186/s12877-026-07398-7 (PMC13188782; doi:10.1186/s12877-026-07398-7)
Supplement: Supplementary file 3 — Supplementary Material 3. [file 12877_2026_7398_MOESM3_ESM.pdf]

Additional File 3

"Deliver phase": Focus Group Guide for family caregivers and professionals – Feedback on the Intervention

Overall Feasibility

- How practical was it for you to participate in the programme (e.g., scheduling, location, duration)?
- Were there any barriers that made attendance or engagement challenging?
- Did the format (group sessions, individual sessions, materials) fit well with your needs and constraints?

Acceptability

- How comfortable did you feel during the sessions?
- Were the content and activities relevant to your experience as a caregiver/professional?
- Did you feel encouraged to participate actively?

Clarity

- Were the objectives of the programme clear from the start?
- Was the information and material presented in an understandable way?
- Were there any aspects of the programme that felt unclear or confusing?

Key Benefits

- What were the most valuable aspects of the programme for you?
- Did you notice any changes in knowledge, skills, emotions, or relationships as a result of the programme?

Suggestions for Improvement

- What would you change or add to make the programme more useful?
- Are there topics or activities that should be included in the future?
