# Supplementary material for: Co-designing a psychoeducational intervention for FCs of institutionalized older adults : a participatory double diamond approach
Source: BMC Geriatr. 2026 Apr 6;26:691. doi: 10.1186/s12877-026-07398-7 (PMC13188782; doi:10.1186/s12877-026-07398-7)
Supplement: Supplementary file 4 — Supplementary Material 4. [file 12877_2026_7398_MOESM4_ESM.pdf]

Additional File 4

Professionals' characteristics

ID	Gender	Age (years)	Professional profile	Experience in geriatrics (years)
P01	Female	32	Nurse	5
P02	Female	34	Psychologist	6
P03	Male	36	Psychologist	7
P04	Female	38	Nurse	8
P05	Female	40	Psychologist	9
P06	Male	42	Psychologist	10
P07	Female	43	Psychologist	10
P08	Female	44	Psychologist	12
P09	Female	45	Nurse	13
P10	Female	46	Physician	15
P11	Female	47	Psychologist	16
P12	Male	48	Nurse	18
P13	Female	50	Psychologist	20
P14	Male	52	Psychologist	18
P15	Female	54	Physician	20
P16	Female	61	Physician	23
P17	Female	65	Physician	25

Family caregivers' characteristics

ID	Gender (Female/male)	Age (years)	Education level
FC01	Female	42	Secondary
FC02	Female	45	Primary
FC03	Female	47	Secondary
FC04	Female	49	No schooling
FC05	Female	50	Primary
FC06	Female	52	Secondary
FC07	Female	54	Tertiary
FC08	Female	55	Tertiary
FC09	Female	56	Primary
FC10	Female	57	Tertiary
FC11	Female	58	Tertiary

ID	Gender (Female/male)	Age (years)	Education level
FC12	Female	59	Tertiary
FC13	Female	60	Secondary
FC14	Female	61	Tertiary
FC15	Female	62	No schooling
FC16	Female	63	Primary
FC17	Female	64	Tertiary
FC18	Female	65	Tertiary
FC19	Female	66	Secondary
FC20	Female	67	Tertiary
FC21	Female	68	Tertiary
FC22	Female	69	Tertiary
FC23	Female	70	Primary
FC24	Female	71	Tertiary
FC25	Female	72	Tertiary
FC26	Female	73	No schooling
FC27	Female	74	Tertiary
FC28	Female	75	Secondary
FC29	Female	76	Tertiary
FC30	Female	77	Tertiary
FC31	Female	78	Tertiary
FC32	Female	79	Tertiary
FC33	Female	80	No schooling
FC34	Female	82	Tertiary
FC35	Female	84	Tertiary
FC36	Female	86	No schooling
FC37	Female	88	Tertiary
FC38	Male	53	Primary
FC39	Male	58	Secondary
FC40	Male	61	Tertiary
FC41	Male	65	Tertiary
FC42	Male	69	Tertiary
FC43	Male	72	Primary
FC44	Male	75	Tertiary
FC45	Male	78	Secondary
